# A meta-analysis of the effects of high-intensity interval training on circulatory system-related indicators in sedentary populations

**DOI:** 10.3389/fphys.2025.1702247

**Published:** 2025-12-10

**Authors:** Guoshuai Li, Depeng Dong

**Affiliations:** 1 School of Physical Education, Shandong University, Jinan, Shandong, China; 2 School of Sports Science, Qufu Normal University, Qufu, Shandong, China

**Keywords:** high-intensity interval training, sedentary population, blood pressure, pulse wave velocity, endothelial function, meta-analysis

## Abstract

**Objective:**

This study systematically evaluated the effects of high-intensity interval training (HIIT) on circulatory indicators in sedentary populations. Following the framework of systematic review and meta-analysis, it synthesized current evidence to support the evidence-based application of HIIT in exercise interventions for sedentary individuals.

**Methods:**

This systematic review and meta-analysis followed PRISMA guidelines and was registered on PROSPERO. We searched PubMed, Web of Science, Embase, and CNKI for studies published between January 2000 and July 2025. Inclusion criteria used the PICOS framework: Participants (sedentary individuals), Intervention (HIIT), Comparison (control), Outcomes (blood pressure, pulse wave velocity, flow-mediated dilation, heart rate), and Study type randomized controlled trials. The search yielded 434 records. After duplicate removal and screening, 14 RCTs (500 participants) were included. The Cochrane Risk of Bias tool assessed study quality, and analyses used RevMan 5.4.

**Results:**

Significant differences were observed between the HIIT and control groups in several outcomes: systolic blood pressure (SBP) (MD = −5.02, 95% CI: −7.29 to −2.76, P < 0.0001), diastolic blood pressure (DBP) (MD = −2.43, 95% CI: −4.08 to −0.79, P = 0.004), pulse wave velocity (PWV) (MD = −0.28, 95% CI: −0.56 to −0.01, P = 0.04), flow-mediated dilation (FMD) (SMD = 1.12, 95% CI: 0.32–1.92, P = 0.006), and heart rate (MD = −0.36, 95% CI: −0.69 to −0.03, P = 0.03). Subgroup analysis of FMD revealed no heterogeneity in studies with a mean participant age of >30 years (P = 0.38, I^2^ = 0%). However, substantial heterogeneity remained in studies with a mean age ≤30 years (P = 0.0003, I^2^ = 79%), suggesting age may be a major source of heterogeneity.

**Conclusion:**

HIIT effectively improves key circulatory indicators in sedentary populations, including blood pressure, vascular elasticity, and endothelial function, making it a valuable exercise strategy for vascular health management. However, further high-quality and standardized clinical trials are needed to confirm its long-term efficacy and safety.

**Systematic Review Registration:**

https://www.crd.york.ac.uk/prospero/display_record.php?, identifier CRD420251106079.

## 1 Introduction

With the rapid progress of modern science and technology, sedentary behavior has become a major global public health concern. Sedentary behavior has significantly contributed to the increase in the incidence of obesity, diabetes and cardiovascular diseases (WHO, 2020). Projections indicate that by 2035, more than half of the world’s population will be overweight or obese (Delfan et al., 2024). Sedentary behavior is defined as any waking activity with an energy expenditure of ≤1.5 metabolic equivalents (METs) performed in a sitting, reclining, or lying posture (WHO, 2020), and it accounts for a substantial part of adults’ daily lives. Numerous epidemiological studies have shown that sedentary behavior is strongly associated with higher risks of chronic diseases, particularly cardiovascular disease and type 2 diabetes (Chen et al., 2025). According to the World Health Organization, over 80% of adolescents and 27.5% of adults worldwide fail to meet recommended physical activity levels, making sedentary behavior a major determinant of global health (WHO, 2024).

The circulatory system, essential for maintaining normal organ function, is highly sensitive to environmental changes. Sedentary behavior can trigger maladaptive responses in the circulatory system, such as impaired endothelial function, increased arterial stiffness, and elevated blood pressure (Shruthi et al., 2024; Silva et al., 2024). Specifically, these responses include elevated systolic and diastolic blood pressure, increased resting heart rate, accelerated pulse wave velocity, endothelial dysfunction, reduced cardiac output, altered arterial diameter, and abnormal levels of vascular endothelial growth factors. These indicators are not only early markers of cardiovascular disease onset and progression but also key outcomes for evaluating intervention effectiveness (Tomiyama, 2023).

In recent years, high-intensity interval training (HIIT) has gained widespread attention as an exercise intervention because of its efficiency, short duration, and ease of implementation. HIIT typically involves alternating bouts of high-intensity exercise with intervals of low-intensity recovery or rest, usually within a short session. Evidence shows that HIIT significantly enhances cardiorespiratory fitness, improves metabolic status, and benefits vascular health (Batacan et al., 2017; Sawyer et al., 2020; Ko et al., 2025). Compared with moderate-intensity continuous training (MICT), HIIT improves maximal oxygen uptake more effectively and shows equal or greater benefits for blood pressure, heart rate, vascular elasticity, and endothelial function (Chin et al., 2020; Poon et al., 2021; Shishira et al., 2024).

For sedentary populations, time constraints and low motivation remain key barriers to exercise interventions. HIIT, characterized by “brief, repeated, and efficient sessions,” provides a feasible strategy for sedentary individuals. Studies suggest that HIIT can improve key circulatory indicators in physically inactive populations, such as systolic and diastolic blood pressure, resting heart rate, pulse wave velocity, and endothelial function (Tang et al., 2022). Moreover, HIIT’s effects on cardiac output, arterial stiffness, arterial diameter, and vascular endothelial growth factors are gaining increasing attention. However, findings remain inconsistent, partly due to small sample sizes and variable study quality.

Although several systematic reviews and meta-analyses have addressed HIIT’s effects on cardiovascular health in general adults or specific patient groups, few have focused on sedentary populations, especially on multiple circulatory outcomes. Sedentary populations have unique health risks, and their vascular responses to exercise interventions may differ from those of the general population. Systematically synthesizing and quantifying HIIT’s effects on circulatory indicators in sedentary populations will enrich the theoretical framework and provide evidence-based guidance for developing more scientific and individualized interventions.

Notably, as understanding of exercise interventions continues to deepen, high-intensity interval training (HIIT)—a time-efficient exercise approach involving alternating short bursts of vigorous activity with brief recovery periods—has been shown to significantly enhance skeletal muscle glucose uptake, improve insulin sensitivity, and upregulate GLUT-4 expression while reducing overall training time (Delfan et al., 2022). Furthermore, investigation into the synergistic effects of HIIT combined with other exercise modalities—such as resistance training—or nutritional interventions—such as spirulina and spinach-derived thylakoids—has become a growing research focus. These integrated approaches are anticipated to yield greater benefits in modulating adipokine activity, enhancing lipid metabolism, and attenuating chronic inflammation (Saeidi et al., 2023a; Saeidi et al., 2023b). Therefore, systematic evaluation of the effects of HIIT on circulatory parameters in sedentary individuals will establish an evidence-based foundation for designing comprehensive health promotion programs that incorporate multiple forms of physical activity, including aerobic, resistance, and flexibility training (Ghanbari-Niaki et al., 2019; Tourny et al., 2023).

Therefore, this study conducts a meta-analysis of randomized controlled trials and intervention studies to systematically evaluate HIIT’s effects on multiple circulatory outcomes in sedentary populations, including systolic and diastolic blood pressure, heart rate, pulse wave velocity, endothelial function, cardiac output, arterial stiffness, arterial diameter, and vascular endothelial growth factors. By quantitatively analyzing HIIT’s benefits and underlying mechanisms, this study provides theoretical evidence and practical guidance for public health policymaking, exercise prescription, and future research.

Building on this premise, the present review aims to address a central research question: Can high-intensity interval training (HIIT) significantly improve key cardiovascular markers in sedentary populations, including blood pressure, arterial stiffness (measured by pulse wave velocity), endothelial function (assessed by flow-mediated dilation), and resting heart rate? The primary objective of this review is to perform a meta-analysis of randomized controlled trials (RCTs) to systematically evaluate the overall impact of HIIT on the aforementioned cardiovascular outcomes in sedentary individuals. Secondly, this review aims to explore how factors such as age influence intervention effects through subgroup analyses and to elucidate the underlying physiological mechanisms. These findings are expected to offer theoretical evidence and practical guidance for public health policy, exercise prescription, and future research.

This study was originally designed to comprehensively evaluate a wide range of circulatory parameters through a systematic search, including cardiac output, arterial stiffness, arterial diameter, and vascular endothelial growth factor (VEGF). However, the final meta-analysis was restricted in scope. Because data were scarce or inconsistently reported for several parameters among the included randomized controlled trials, reliable statistical analyses were feasible only for five outcomes with sufficient and consistent data: systolic blood pressure (SBP), diastolic blood pressure (DBP), heart rate (HR), pulse wave velocity (PWV), and flow-mediated dilation (FMD).

## 2 Materials and methods

This meta-analysis followed the PRISMA 2020 Statement: Updated Guidelines for Reporting Systematic Reviews (Page et al., 2021), and the protocol was registered on the PROSPERO platform (CRD420251106079).

### 2.1 Data sources

#### 2.1.1 Researchers

Two researchers (the first and second authors) independently and blindly conducted the literature search to ensure objectivity and consistency in study selection.

#### 2.1.2 Databases

The search encompassed major Chinese and English databases, including CNKI, Wanfang, PubMed, Web of Science, and Embase.

#### 2.1.3 Search terms

The English search terms were: (“High-Intensity Interval Training” OR “HIIT” OR “Interval Training”) AND (“Sedentary Population” OR “Sedentary Behavior” OR “Sedentary Lifestyle” OR “Sedentary Adults”) AND (“Circulatory System” OR “Cardiovascular System” OR “Cardiovascular Health” OR “Vascular Function” OR “Blood Pressure” OR “Heart Rate” OR “Arterial Stiffness” OR “Blood Lipids”). The Chinese search terms were: (“高强度间歇训练” OR “高强度间歇运动” OR “HIIT” OR “间歇运动”) AND (“久坐” OR “久坐行为” OR “久坐人群” OR “久坐生活方式”) AND (“血液循环系统” OR “心血管系统” OR “血管功能” OR “心血管健康” OR “血压” OR “心率” OR “动脉硬化” OR “血脂”).

#### 2.1.4 Search period

The search period extended from 1 January 2000 to 10 July 2025.

#### 2.1.5 Search strategy

**FIGURE 1:**
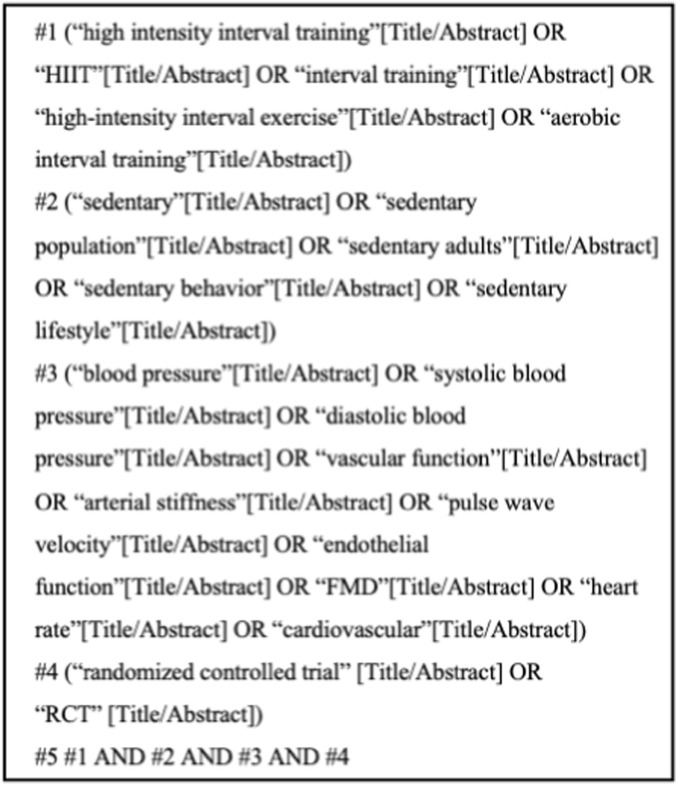
Search strategy diagram for PubMed database.

The PubMed search strategy is provided as an example (Figure 1).

### 2.2 Inclusion and exclusion criteria

The inclusion and exclusion criteria were defined using the PICOS framework of evidence-based medicine: Population, Intervention, Comparison, Outcomes, and Study design. Two researchers independently conducted the screening process. Disagreements were resolved through discussion, and a third researcher adjudicated unresolved issues.

#### 2.2.1 Inclusion criteria

① Participants: Individuals of any nationality, region, or sex who met the definition of sedentary behavior were eligible. Sedentary behavior was defined as any waking activity with an energy expenditure of ≤1.5 METs performed in a sitting, reclining, or lying posture (WHO, 2020). ② Intervention: High-Intensity Interval Training (HIIT), defined as alternating bouts of high-intensity exercise (typically 80%–95% of maximal heart rate or ≥80% of maximal oxygen uptake) with periods of low-intensity active or passive recovery. High-intensity bouts generally lasted seconds to a few minutes, with recovery intervals of similar or slightly longer duration. Total sessions typically lasted 10–30 min, aiming to elevate heart rate and oxygen consumption and to induce greater metabolic, cardiovascular, and neuromuscular adaptations (Gibala and McGee, 2008; ACSM, 2021). ③ Comparison: Control interventions were not restricted, with no specific requirements for frequency or duration. ④ Outcomes: Pre- and post-intervention measures of systolic blood pressure (SBP), diastolic blood pressure (DBP), pulse wave velocity (PWV), flow-mediated dilation (FMD), and heart rate (HR). ⑤ Study Design: Only randomized controlled trials (RCTs) were included.

#### 2.2.2 Exclusion criteria

Studies were excluded if they met any of the following criteria: non-Chinese or non-English publications, duplicate reports, animal experiments, non-randomized or non-parallel design studies, acute trials, studies involving non-sedentary populations, studies not using HIIT as the main intervention, or studies lacking full-text availability or valid outcome data.

### 2.3 Literature screening and data extraction

**FIGURE 2:**
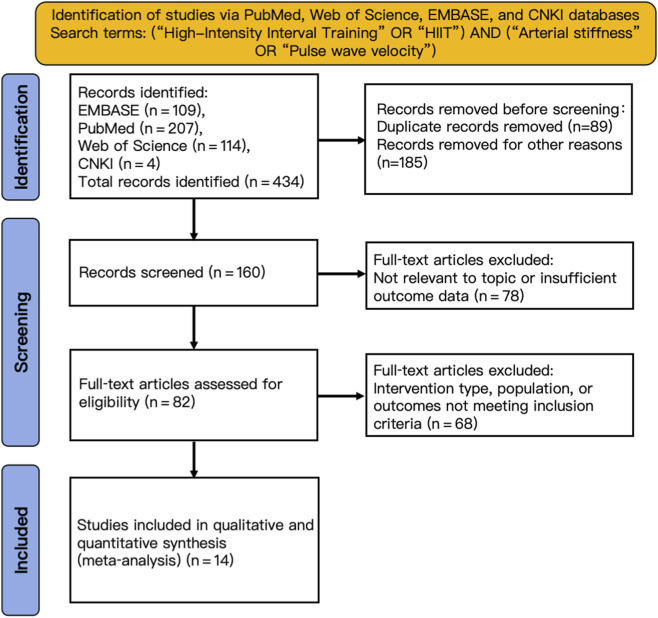
PRISMA flow diagram of the study selection process.

A total of 434 Chinese and English articles were imported into EndNote 20 for duplicate detection and removal. Two reviewers independently screened studies and extracted data, cross-checking results for consistency. Discrepancies were resolved through discussion, with a third reviewer making the final decision if needed. For missing data, the original authors were contacted to obtain supplementary information. A standardized extraction form was developed and systematically applied to ensure consistency across all included studies. The literature screening process strictly followed the PRISMA 2020 statement (Page et al., 2021), and the screening process is detailed in Figure 2. Screening consisted of preliminary review, title and abstract screening, and full-text evaluation. Ultimately, 14 studies on HIIT and circulatory indicators in sedentary populations met the inclusion criteria, comprising 500 participants (Table 1 for study characteristics).

**TABLE 1:** Basic characteristics of included literature.

Included in the study	Subject population	Intervention measures/sample size/gender ratio (male: female)	Sample age (y)/weight (kg)	Intervention plan		Duration and frequency	Primary findings
				Intervention group	Control group		
Afousi et al. (2018)	Patients with type 2 diabetes for at least 2 years, with a glycated hemoglobin (HbA1c) level >6%, aged 45–60 years, with a history or current hypertension, and who have not undergone exercise training in the past 6 months.	LV-HITT/18/9:9 Control group/17/9:8	LV-HIIT: 54.78 ± 6.19/85.38 ± 11.23 Control group: 54.24 ± 5.61/82.23 ± 10.58	LV-HIIT: 12 sets of 1.5-min high-intensity cycling (85%–90% HRmax) alternated with 2-min low-intensity recovery (55%–60% HRmax).	Maintain daily activities without any exercise intervention.	12 weeks, 3 times a week	The 12-week LV-HIIT significantly increased FMD (from 3.45% ± 0.97% to 7.39% ± 3.6%, P < 0.005), which was superior to moderate-intensity continuous training (CMIT) (from 3.83% ± 1.13% to 4.81% ± 2.36%). It also increased the forward shear rate (+38%), decreased the reverse shear rate (−22%), and reduced OSI (−15%), and significantly elevated the NOx level (+53%). The CMIT group only slightly improved FMD and did not significantly change the shear pattern.
Chen et al. (2024)	Female college students aged between 19 and 26, with the right leg as the dominant side, experiencing two or more mild to moderate symptoms of COVID-19, and classified as having a low level of physical activity based on the International Physical Activity Questionnaire (IPAQ), who are sedentary.	HITT/12/0:12 Control group/12/0:12	HIIT: 24.0 ± 1.04/66.0 ± 9.15 Control group: 23.75 ± 0.75/64.15 ± 8.72	HIIT: 4 sets of 4-min running (75%–80% of HRmax), with 4-min rest between each set.	Maintain the habit of sitting still (for at least 6 h per day)	Once a week, twice a day	Cold environment HIIT is superior: Significantly improves baPWV (effect size d = 1.24) and cerebral hemodynamic (HbO ↑ 38.8%), with better effects than the normal temperature group; Ankle-brachial pulse wave velocity (baPWV): The cold environment group significantly decreased by 11% (p < 0.05), while the normal temperature group showed no change; Pulse pressure (PP): The cold environment group significantly decreased (p < 0.05), while the control group significantly increased; Cerebral hemodynamics: Oxygenated hemoglobin (HbO): The cold environment group significantly increased in the left M1 (+38.8%) and right DLPFC (+29.7%) (p < 0.05) Total hemoglobin (HbT): The cold environment group significantly increased in the left PMC/SMC (+32.1%) and right M1 (+28.9%); Body composition and balance ability: Body fat percentage: The cold environment group significantly decreased (p < 0.05) Y balance test (YBTs): The cold environment group scored significantly higher (p < 0.01)
Chidnok et al. (2020)	Healthy women aged 18–26, who do not engage in regular physical exercise, with no previous history of cardiovascular diseases or other metabolic disorders.	HIIT/11/0:11 Control group/11/0:11	Age: 21.27 ± 0.703 Weight: HIIT: 55.73 ± 12.11 Control group:55.18 ± 12.4	HIIT: Perform 5 1-min high-intensity interval training (HITT) sessions at 80%–85% of maximum heart rate on a power bike, with a 2-min rest period between each session.	Keep your original eating habits unchanged and maintain the current level of daily activity.	6 weeks, 3 times a week	Blood flow-mediated dilation (FMD): The HIIT group showed a significant improvement (9.26% → 14.01%, p < 0.05), with an effect size of d = 0.82; Central artery-ankle vascular index (CAVI): The HIIT group significantly decreased (6.39 → 5.91, p < 0.05), reflecting an improvement in arterial stiffness; Maximum oxygen uptake (VO_2_max): The HIIT group increased by 21% (20.10 → 24.34 mL/kg/min, p < 0.05)
Collins et al. (2023)	Healthy men who sit for long periods of time (exercise less than 2 days per week, and have no planned exercise in the past 12 months).	P-HIT/15/15:0 Control group/14/14:0	P-HIT: 47.3 ± 5.1/94.8 ± 20.2 Control group: 51.2 ± 7/89.4 ± 12.1	P-HIT: 30 s of high-intensity interval training (at 85% of maximum heart rate) + 2.5 min of passive recovery (increasing from 4 sets per session to 10 sets per session, with one additional set every 2 weeks)	Keep your original eating habits unchanged and maintain the current level of daily activity.	12 weeks, 3 times a week	After 12 weeks of intervention, all exercise groups (MICT/P-HIT/A-HIT) significantly increased VO_2_ by 85% (↑3.1–5.1 mL/kg/min, p < 0.001), FMD (↑3.4%–4.9%, p < 0.05), and exercise duration (↑62–90 s, p < 0.001). The SDNN (a HRV indicator) in the MICT and P-HIT groups significantly improved (↑7–14 ms, p ≤ 0.05), but there was no change in the A-HIT group. Active recovery (A-HIT) did not show additional benefits.
Deiseroth et al. (2019)	Those aged between 50 and 80 years old, who have two or more cardiovascular risk factors or are current smokers.	HIIT/38/20:18 Control group/30/11:19	58 ± 6/95.5 ± 13.9	HIIT: Based on Nordic walking, it consists of 4 sets of 4-min high-intensity intervals (80%–90% HRmax) interspersed with 3-min active recovery (60%–70% HRmax). The intensity is gradually increased over the first 2 weeks to help sedentary participants adapt to the exercise load, and then it is carried out officially for the next 10 weeks.	Only accept the standard recommendations for physical activity.	12 weeks, 3 times a week	⟷ 12-week HIIT did not improve PWV (intervention group: 8.1 → 8.1 m/s vs. control group: 8.2 → 7.9 m/s, p = 0.60); ↑ VO_2_max significantly increased (intervention group: 26.4 → 28.6 mL/min/kg, p < 0.001); ⟷ Systolic blood pressure showed no significant change (intervention group: 133 → 134 mmHg)
Van den Eynde et al. (2020)	Those aged between 50 and 80 years old, who have two or more cardiovascular risk factors or are current smokers.	HIIT/34/- Control group/40/-	58.7 ± 6.6/-	HIIT: Based on Nordic walking, 4 sets of 4-min high-intensity intervals (80%–90% HRmax) interspersed with 3-min active recovery (60%–70% HRmax), gradually increasing the intensity. The first week is the adaptation period (75% HRmax), and from the second week onwards, the intensity is gradually increased to the maximum (80%–90% HRmax).	Follow the corresponding exercise recommendations as outlined in the “European Guidelines for the Prevention of Cardiovascular Diseases”.	12 weeks, 3 times a week	The protein-bound CML level in the healthy and active group (HA) was significantly higher than that in the sedentary group (HS: 3.1 ± 0.5 vs. 3.6 ± 0.6 μmol/L, p < 0.05) and the high-risk sedentary group (SR: 2.6 ± 0.5 μmol/L). VO_2_max was positively correlated with CML (β = 0.48, 95% CI: 0.34–0.62). After 12 weeks of HIIT intervention, although the SR group improved their cardio-pulmonary function (VO_2_max ↑ 2.3 mL/min/kg, p < 0.05), all glycation indicators showed no significant changes (p > 0.05).
Kim et al. (2017)	55–79 years old, without a history of smoking, without significant cardiovascular diseases and other major clinical disorders (such as diabetes, liver disease and kidney disease). Before enrollment, they had not undergone regular aerobic exercise training for at least 12 months (that is, no more than 2 times per week, each session less than 30 min of aerobic exercise).	HIIT/14/-Control group/11/-	HIIT: 65 ± 1/72.9 ± 4.0 Control group: 63 ± 2/68.2 ± 4.3	HIIT: 10 min of warm-up at 70% peak heart rate, using a full-body non-weight-bearing air braking treadmill, upper body pulling/pushing handlebars, lower body cycling, 4 min per set, repeat 4 times, 3 min of rest between sets, 5 min of cool-down.	Maintain the previous habit of prolonged sitting.	8 weeks, 4 times a week	⟷ Pulse wave velocity: Only the MICT group showed a significant improvement of −0.5 m/s. There were no significant changes in the HIIT group and the CONT group. ⟷ Blood pressure: Both the aortic blood pressure and the brachial artery blood pressure showed no significant changes.
Huang et al. (2025)	Participants who sit for long periods, have irregular exercise habits, do not have cardiovascular diseases, are not overweight, do not suffer from color blindness, and do not have cognitive impairments.	HIIT/16/8:8 Control group/16/7:9	HIIT: 21.44 ± 1.93/60.39 ± 7.39 Control group: 22.19 ± 1.52/62.52 ± 11.02	HIIT: On the power bike, perform 1 min of high-intensity exercise (at 90% VO_2_max) followed by 1 min of low-intensity exercise (at 50% VO_2_max). Each set lasts for 2 min, and this is repeated for 10 sets.	Maintain the previous habit of prolonged sitting.	8 weeks, 3 times a week	↓cfPWV: HIIT and MICT reduced; ↓FMD: HIIT and MICT significantly improved, while RE showed only a slight improvement;
Jalaludeen et al. (2020)	Healthy male and female participants who lack physical exercise.	HIIT/21/-Control group/20/-	HITT:21 ± 1.7/73.9 ± 14.4 Control group: 22 ± 3.5/74.3 ± 15.9	On the Wattbike training device, perform three sets of 30-s all-out cycling (with a resistance of 7.5% of body weight), followed by a 2-min rest period without load.	Maintain the previous level of physical activity and dietary habits.	4 weeks, 3 times a week	Four weeks of high-intensity interval training (HIIT) significantly improved the mechanical function of the aortic lumen (reserve period strain ↑13.9%, p = 0.033; conductive period strain ↑8.9%, p = 0.023), the aortic dilatation (↑2.1 cm^2^·dyn^−1^·10^−3^, p = 0.031), and reduced the aortic stiffness index (↓2.6, p = 0.041). The changes in aortic stiffness were significantly correlated with aortic dilatation (*R* ^2^ = 0.613, p = 0.002).
Shenouda et al. (2017)	Men aged 19–44, who usually sit for long periods but are in good health.	SIT/9/9:0 Control group/6/6:0	SIT: 27 ± 7/84 ± 23 Control group: 26 ± 8/78 ± 25	SIT: Resistance load 5% of body weight. Perform all-out sprint cycling for 3 sets, each set lasting 20 s, with 3-min rest between sets.	Maintain the previous habit of prolonged sitting.	12 weeks, 1 time in the 1st week, 2 times in the 2nd week, 3 times each week thereafter, with only 1 time in the 7th week.	Vascular endothelial function FMD: In the MICT group: It significantly increased (↑2.2%) after 6 weeks, but returned to the baseline level at 12 weeks; In the SIT group: There was no significant change, and there was a downward trend at 12 weeks (↓1.8%) ⟷ PWV: There were no significant changes in all groups.
Streese et al. (2020)	Participants who had a history of prolonged sitting and were aged between 50 and 80 years old, with at least 2 cardiovascular risk factors.	HITT/36/20:16 Control group/33/14:19	HITT: 58 ± 5/95.6 ± 12.3 Control group: 58 ± 7/92.9 ± 14.6	HIIT group: 10 min of warm-up (60%–70% HRmax) → 4 sets of 4-min high-intensity intervals (80%–90% HRmax) → 3 min of active recovery (60%–70% HRmax) → 10 min of cool-down	Carry out regular physical activities in accordance with European guidelines (at least 30 min per day at moderate intensity or 15 min per day at high intensity)	12 weeks, 3 times a week	↑The maximum dilation amplitude (ADmax) of the retinal arterioles in the HIIT group significantly improved (2.7% → 3.0%, p = 0.027), while there was no change in the Control group; ↑The area under the arterial dilation curve (AFarea) increased (32.6 → 37.7%*s, p = 0.034), and was significantly correlated with the increase in VO_2_peak (r = 0.12%/mL/min/kg); There was no direct correlation between the improvement of classic cardiovascular risk factors (BMI, LDL, etc.) and the changes in microvascular function; The reactivity of venous vessels (VDmax/VFarea) showed no significant change.
Sun et al. (2024)	A student from Nanjing Agricultural University who sits for long periods, is healthy and has a normal weight.	HIIT/6/-Control group/6/-	Age: 18.5 ± 0.3 Weight: HIIT: 64.4 ± 5.1 Control group: 53.5 ± 8.5	HITT: The intensity should be 85%–95% of the maximum heart rate. For the first to fourth weeks: 8-s all-out sprint followed by 24 s of active recovery walking. For the fifth to eighth weeks: 10-s all-out sprint followed by 30 s of active recovery walking. Complete 21 to 24 repetitions within 20 min.	Maintain the previous level of physical activity and dietary habits.	8 weeks, 3 times a week	The systolic and diastolic blood pressures in the HITT group were significantly reduced.
Toohey (2018)	People who are sedentary and in the later stage of the “Whole Body Activity for Cancer Patients” (PEACE) treatment model within 24 months after cancer diagnosis.	LV-HIIT/24/0:24 Control group/12/0:12	LV-HIIT: 48 ± 11.9/48 ± 11.9 Control group: 57 ± 11.5/57 ± 11.5	LV-HIIT: On a stationary bicycle, ride for 30 s (at ≥ 85% predicted maximum heart rate), repeat 7 times, with 60 s of rest between each set. In the first week, do 3 sets, then increase by 1 set each week until the 5th week when all 7 sets are completed.	Maintain your previous lifestyle.	12 weeks, 3 times a week	↓ In terms of cardiovascular function: The LVHIIT group showed a 4.9% decrease in central systolic pressure (CSP) and a 7.96% decrease in pulse pressure (PP) (medium effect size); ↓ SBP, DBP, HR, and PWV all decreased slightly, but there were no differences between the groups.
Tucker et al. (2021)	Overweight or obese men aged 18–50 with no smoking history, who currently lack physical activity but are capable of engaging in vigorous exercise; have not taken any dietary supplements or medications for cardiovascular diseases, hypertension or diabetes; and are willing to consume two doughnuts every day for 6 days a week within 4 weeks.	HITT/10/10:0 Control group/9/9:0	HITT: 30 ± 7/96.0 ± 7.7 Control group: 28 ± 9/93.1 ± 11.8	Dietary intervention: 2 muffins per day (total 48/4 weeks), additional intake of 14,500 kcal, 600 kcal per day (70% fat + sugar), total carbohydrates 1,686 g, fat 815 g Exercise intervention: 1 min of high-intensity cycling (90%–95% HRmax) + 1 min of rest	Dietary intervention: 2 doughnuts per day (total 48/4 weeks), additional intake of 14,500 kcal, 600 kcal per day (70% fat + sugar), total carbohydrates 1,686 g, fat 815 g. Exercise intervention: Maintain the previous sedentary lifestyle.	4 weeks, 4 times a week	↑ Weight change: The control group showed no change, while the HIIT group increased by 1.2 kg (p = 0.02) ↑ Visceral fat: The HIIT group increased by 6.4% (p = 0.04), while other groups showed no change ↓ Energy compensation: The control group spontaneously reduced 239 kcal per day (p = 0.05), offsetting 84% of the calorie intake from doughnuts ↓ Glucose response: The control group’s OGTT glucose iAUC decreased by 25.6% (p = 0.001) Insulin sensitivity: There were no significant changes in HOMA-IR, Matsuda index, etc. Endothelial function (FMD): No significant changes Blood pressure: Systolic/diastolic blood pressure remained stable VO_2_max: The HIIT group increased by 12.1% (p < 0.001), and the MICT group increased by 9.2% (p = 0.001)

Extracted data included: study characteristics (first author, year), participant characteristics (sex, age), intervention details (protocol, duration, frequency), sample size, sex ratio, mean age, and body weight. Primary outcomes included SBP, DBP, HR, PWV, FMD, cardiac output (CO), and arterial stiffness (AS).

### 2.4 Quality assessment

The methodological quality of included studies was independently assessed by two researchers using the Cochrane risk of bias tool. A third researcher resolved disagreements. The assessment covered randomization, allocation concealment, blinding, data completeness, selective reporting, and other potential biases.

### 2.5 Outcome measures

The primary outcomes were systolic blood pressure (SBP), diastolic blood pressure (DBP), pulse wave velocity (PWV), flow-mediated dilation (FMD), and heart rate (HR). Among these, SBP, DBP, PWV, and FMD were classified as vascular function-related indicators.

### 2.6 Statistical analysis

RevMan 5.4 software was used for data synthesis, subgroup analyses, and the creation of forest plots. Data from all included studies were systematically extracted and analyzed. The mean difference was computed as: $M_{change}=M_{final}-M_{baseline}$ ), and the standard deviation of the difference was computed as: ${SD}_{change}=\sqrt{{SD}_{baseline}^{2}+{SD}_{final}^{2}-2\times R\times {SD}_{baseline}\times {SD}_{final}}$ , where R represents the correlation coefficient, $M_{baseline}$ represents the average value of the pre-test, $M_{final}$ represents the average value of the post-test, and SD as defined above, with R = 0.5 (Higgins and Deeks, 2023). Because the original individual-level data were unavailable, this study assumed a correlation coefficient of R = 0.5, a commonly used value for calculating the standard deviation of change scores. This assumption is generally regarded as reasonable in studies involving physiological parameters. However, varying the correlation coefficient (e.g., R = 0.3 or R = 0.7) may slightly influence the estimated variance without affecting the overall interpretation of the findings. For continuous variables, MD was used when measurement units were consistent across studies; otherwise, standardized mean difference (SMD) was applied (Zhang et al., 2025). All outcomes were continuous measures. Systolic blood pressure (mmHg), diastolic blood pressure (mmHg), pulse wave velocity (m/s), and heart rate (bpm) were consistently reported across studies and expressed as mean differences. Flow-mediated dilation (FMD) was reported as a standardized mean difference because of inconsistent units across studies.

Heterogeneity was assessed using the Q test and the I^2^ statistic. Conventionally, I^2^ <25% indicates low heterogeneity, 25%–50% indicates moderate heterogeneity, and >50% indicates substantial heterogeneity (Higgins and Deeks, 2023). Given the potential for clinical and methodological diversity among included studies, a random-effects model was employed as the primary analytical approach for all meta-analyses to provide more conservative and generalizable estimates. A fixed-effects model was considered only when heterogeneity was negligible (I^2^ = 0%). When significant heterogeneity was detected, subgroup or sensitivity analyses were conducted to explore potential sources; if unresolved, descriptive analysis was performed. Statistical Significance of pooled effect sizes was set at α = 0.05. Publication bias was assessed using funnel plots.

## 3 Results

As outlined in the Methods section, this review systematically identified studies that reported various cardiovascular parameters. However, as detailed in Section 3.1, sufficient data were available for meta-analysis only for five key indicators: systolic blood pressure (SBP), diastolic blood pressure (DBP), pulse wave velocity (PWV), flow-mediated dilation (FMD), and heart rate (HR). Therefore, the quantitative results presented in this section focus exclusively on these indicators. Data for other prespecified indicators—such as cardiac output, arterial diameter, and vascular endothelial growth factor—were too limited in the included studies to permit meaningful quantitative or descriptive synthesis.

### 3.1 Search results and study characteristics

Fourteen studies were included in the meta-analysis (Table 1), evaluating the effects of high-intensity interval training (HIIT) on multiple cardiovascular outcomes in sedentary populations. Primary outcomes were systolic and diastolic blood pressure, whereas secondary outcomes included pulse wave velocity, endothelial function, and resting heart rate (Figure 2). Notably, despite employing a comprehensive search strategy, the number of studies reporting data on cardiac output, arterial diameter, and vascular endothelial growth factor was insufficient for quantitative synthesis (meta-analysis). The included studies mainly focused on five parameters analyzed in this meta-analysis: systolic blood pressure, diastolic blood pressure, pulse wave velocity, flow-mediated vasodilation, and heart rate. Accordingly, the subsequent analysis and discussion in this study concentrate on these parameters with sufficient data.

### 3.2 Characteristics of HIIT protocols included in the studies

To provide a comprehensive overview of the intervention protocols, this study summarizes the key parameters of the HIIT regimens employed in the 14 included randomized controlled trials (Table 2). The summarized parameters include exercise modality, duration and intensity of the high-intensity phase, duration and pattern of the recovery phase, total session duration, training frequency, and overall intervention period. Importantly, the HIIT protocols included in the studies exhibited substantial variation in exercise modality, intensity, interval duration, recovery method, and intervention duration, which may represent potential sources of heterogeneity in this meta-analysis.

**TABLE 2:** Characteristics of HIIT protocols included in the meta-analysis.

Inclusion in the study (first author, year)	Motor pattern	High-intensity stage		Intermittent stage		Number of repetitions in a single set	Total training duration (in minutes)	Weekly frequency (times)	Total period (weeks)
		Burning time	Intensity	Burning time	Pattern				
Afousi et al. (2018)	Cycle ergometers	90 s	85%–90% HRmax	2 min	Low-intensity cycling	12	∼41	3	12
Chen et al. (2024)	Running	4 min	75%–80% HRmax	4 min	Rest	4	∼42	2	1
Chidnok et al. (2020)	Cycle ergometers	1 min	80%–85% HRmax	2 min	Rest	5	∼25	3	6
Collins et al. (2023)	Cycle ergometers	30 s	85% HRmax	2 min and 30 s	Passive restoration	4∼10	∼20–35	3	12
Deiseroth et al. (2019)	Nordic Walking	4 min	80%–90% HRmax	3 min	Take the initiative to restore	4	∼39	3	12
Van den Eynde et al. (2020)	Nordic Walking	4 min	80%–90% HRmax	3 min	Take the initiative to restore	4	∼40	3	12
Kim et al. (2017)	All-extremity non-weight-bearing air-braked ergometer	4 min	80%–90% HRmax	3 min	Rest	4	∼38	4	8
Huang et al. (2025)	Cycle ergometers	1 min	90% VO_2_max	1 min	50% VO_2_max	10	∼30	3	8
Jalaludeen et al. (2020)	Wattbike	30 s	Full-speed sprint (with a 7.5% weight resistance)	2 min	Rest without load	3	∼18	3	4
Shenouda et al. (2017)	Cycle ergometers	20 s	Full-speed sprint (with a 5% weight resistance)	3 min	Rest	3	∼20	1∼3	12
Streese et al. (2020)	Nordic Walking	4 min	80%–90% HRmax	3 min	Take the initiative to restore	4	∼39	3	12
Sun et al. (2024)	Running	8∼10 s	85%–95% HRmax	24–30 s	Take the initiative to resume walking	21∼24	∼20	3	8
Toohey (2018)	Cycle ergometers	30 s	≥85% HRmax	1 min	Rest	3∼7	∼20	3	12
Tucker et al. (2021)	Cycle ergometers	1 min	90%–95% HRmax	1 min	Rest	8∼11	∼29	4	4

### 3.3 Study characteristics and quality assessment

**FIGURE 3:**
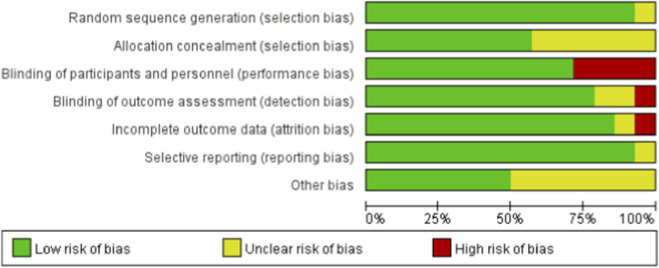
Literature quality evaluation chart included.

Risk of bias was assessed using the Cochrane risk of bias tool, and the overall distribution is shown in Figure 3.

### 3.4 Meta-analysis results

#### 3.4.1 Effects of high-intensity interval training on vascular function indicators

##### 3.4.1.1 Systolic blood pressure (SBP)

**FIGURE 4:**
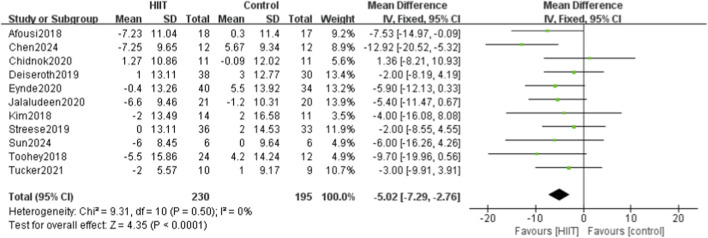
Meta-analysis forest plot of the effects of HIIT intervention (experimental group) and the blank control group on systolic blood pressure.

Eleven of the 14 studies compared HIIT with a control group for systolic blood pressure. Heterogeneity testing showed P = 0.50 and I^2^ = 0%, indicating no heterogeneity; thus, a fixed-effects model was used. Meta-analysis showed that SBP was significantly lower in the HIIT group than in the control group (MD = −5.02, 95% CI: −7.29 to −2.76, P < 0.0001) (Figure 4).

##### 3.4.1.2 Diastolic blood pressure (DBP)

**FIGURE 5:**
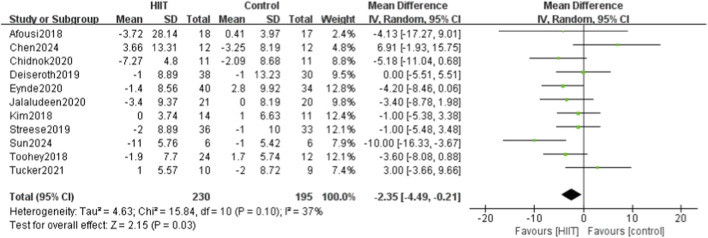
Meta-analysis forest plot showing the effects of HIIT intervention (experimental group) and the blank control group on diastolic blood pressure.

Eleven of the 14 studies compared HIIT with a control group for diastolic blood pressure. Heterogeneity testing showed P = 0.10 and I^2^ = 37%, indicating moderate heterogeneity. Accordingly, a random-effects model was applied. Meta-analysis showed that DBP was significantly lower in the HIIT group than in the control group (MD = −2.35, 95% CI: −4.49 to −0.21, P = 0.03) (Figure 5).

##### 3.4.1.3 Pulse wave velocity (PWV)

**FIGURE 6:**
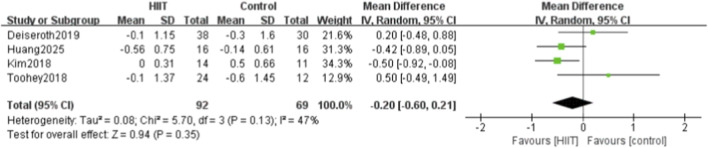
Meta-analysis forest plot showing the effects of HIIT intervention (experimental group) and the blank control group on pulse wave velocity.

Four of the 14 studies compared HIIT with a control group for pulse wave velocity. Heterogeneity testing showed P = 0.13 and I^2^ = 47%, indicating moderate heterogeneity. Therefore, a random-effects model was used. The meta-analysis showed that the reduction in PWV in the HIIT group was not statistically significant (MD = −0.20, 95% CI: −0.60 to 0.21, P = 0.35) (Figure 6).

##### 3.4.1.4 Flow-mediated dilation (FMD)

**FIGURE 7:**
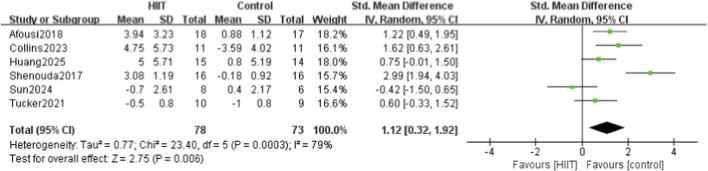
Meta-analysis forest plot showing the effects of HIIT intervention (experimental group) and the blank control group on vascular endothelial function (FMD).

Six of the 14 studies compared HIIT with a control group for endothelial function. Heterogeneity testing showed P = 0.0003 and I^2^ = 79%, indicating high heterogeneity; thus, a random-effects model was used. Meta-analysis showed that HIIT significantly improved FMD (SMD = 1.12, 95% CI: 0.32–1.92, P = 0.006) (Figure 7).

#### 3.4.2 Effects of high-intensity interval training on heart rate

**FIGURE 8:**
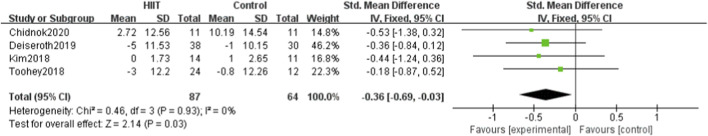
Meta-analysis forest plot showing the effects of HIIT intervention (experimental group) and the blank control group on heart rate.

Resting Heart Rate (HR): Four of the 14 studies compared HIIT with a control group for resting heart rate. Heterogeneity testing showed P = 0.93 and I^2^ = 0%, indicating no heterogeneity; thus, a fixed-effects model was used. Meta-analysis showed that resting HR was significantly lower in the HIIT group than in the control group (MD = −0.36, 95% CI: −0.69 to −0.03, P = 0.03) (Figure 8).

### 3.5 Subgroup analysis results

**FIGURE 9:**
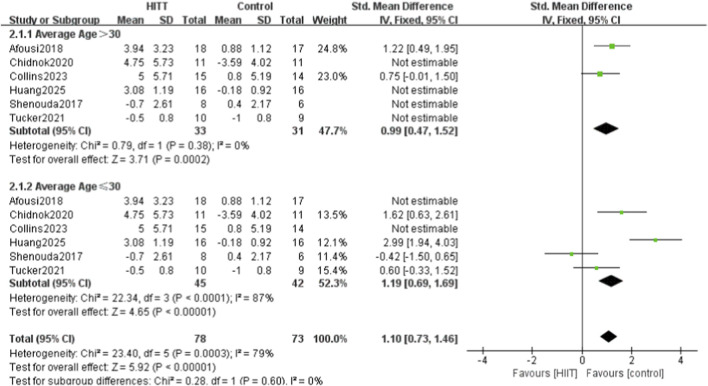
Meta-analysis forest plot of subgroup analysis of vascular endothelial function by different age groups.

For flow-mediated dilation (FMD), variation in participant age may be a key source of heterogeneity. Six studies reporting FMD outcomes were stratified into two subgroups—mean age >30 years and mean age ≤30 years—for further analysis (Figure 9). Two studies included participants with a mean age of>30 years, and four included participants with a mean age of ≤30 years. The >30 years subgroup showed no heterogeneity (P = 0.38, I^2^ = 0%), whereas the ≤30 years subgroup showed high heterogeneity (P = 0.0003, I^2^ = 79%), suggesting that age may be a major source of heterogeneity (Figure 9).

### 3.6 Publication bias analysis

**FIGURE 10:**
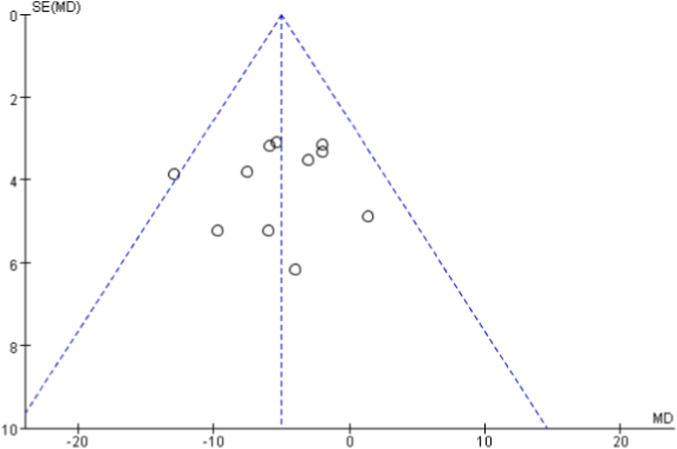
Funnel plot of publication bias based on systolic blood pressure as the outcome indicator.

**FIGURE 11:**
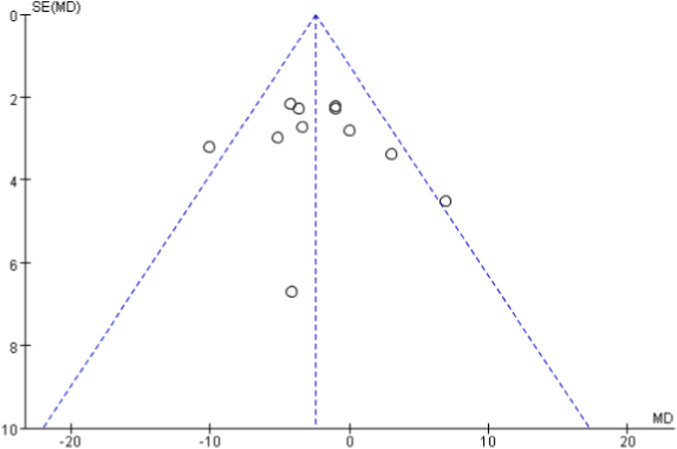
Funnel plot of publication bias based on diastolic blood pressure as the outcome indicator.

**FIGURE 12:**
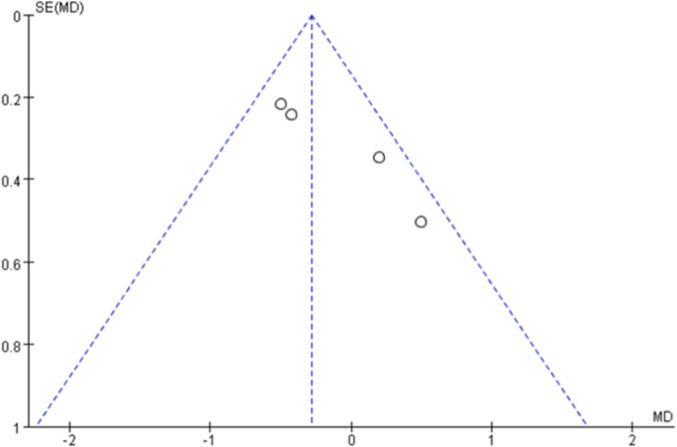
Funnel plot of publication bias based on the outcome measure of pulse wave velocity.

**FIGURE 13:**
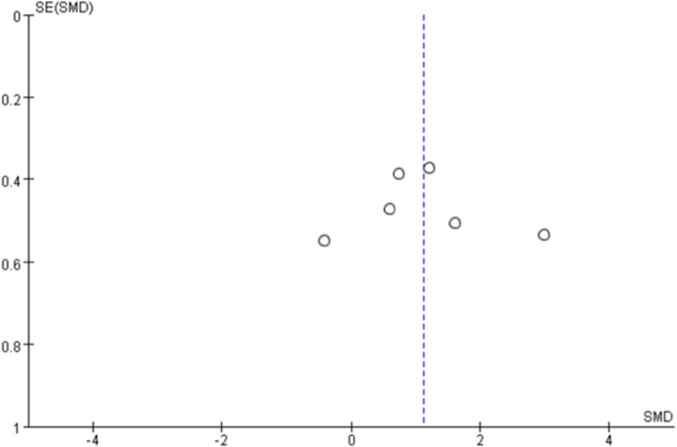
This figure shows the publication bias funnel plot with vascular endothelial function as the outcome indicator.

**FIGURE 14:**
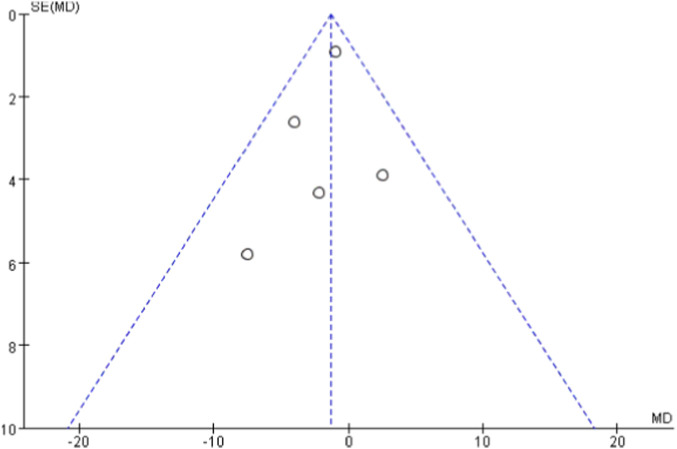
Funnel plot of publication bias based on heart rate as the outcome indicator.

Funnel plots were generated for each outcome to assess publication bias (Figures 10–14). The plots showed an approximately symmetric distribution, indicating no obvious publication bias or other systematic bias.

### 3.7 Sensitivity analysis

**FIGURE 15:**
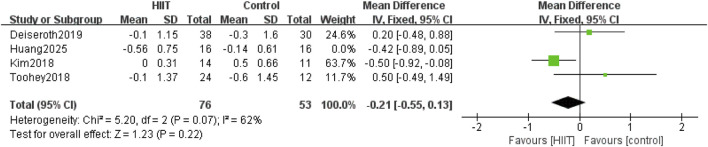
Meta-analysis forest plot (sensitivity analysis) of the effects of HIIT intervention (experimental group) and the blank control group on pulse wave velocity.

To test the robustness of the primary findings, sensitivity analyses were performed by sequentially excluding each of the 14 studies and re-pooling the data. The analyses showed that the effects of HIIT on SBP, DBP, FMD, and HR remained consistent after sequential exclusions, indicating stable and reliable results. For PWV, excluding the study by Huang et al. (2025) changed heterogeneity from moderate (P = 0.13, I^2^ = 47%) to high (P = 0.07, I^2^ = 62%), indicating a notable influence on the overall effect size (Figure 15).

## 4 Discussion

### 4.1 Summary of evidence

Fourteen studies were included, systematically evaluating the effects of high-intensity interval training (HIIT) on key circulatory indicators in sedentary populations, including systolic blood pressure (SBP), diastolic blood pressure (DBP), pulse wave velocity (PWV), flow-mediated dilation (FMD), and heart rate (HR). The meta-analysis showed that HIIT significantly reduced SBP and DBP, enhanced FMD, and had a modest effect on HR. However, when a more conservative random-effects model was applied to account for moderate heterogeneity, the reduction in PWV was no longer statistically significant. Specifically, 11 studies reported that HIIT significantly reduced SBP (MD = −5.02, 95% CI: −7.29 to −2.76), and 11 showed significant reductions in DBP (MD = −2.35, 95% CI: −4.49 to −0.21). Four studies found that HIIT effectively reduced PWV (MD = −0.20, 95% CI: −0.60 to 0.21). Six studies demonstrated significant improvements in FMD (SMD = 1.12, 95% CI: 0.32–1.92), indicating enhanced vascular function. For HR, five studies showed a modest reduction after HIIT (MD = −0.36, 95% CI: −0.69 to −0.03).

Collectively, these findings suggest that HIIT provides multiple benefits for circulatory indicators in sedentary populations, with particularly strong effects on blood pressure, vascular elasticity, and endothelial function. These results offer empirical support for HIIT as an effective strategy to improve cardiovascular health in sedentary individuals.

The inconsistencies observed in the findings across studies can be partly explained by the methodological limitations of the included trials, as identified by our Cochrane risk-of-bias assessment (see Section 2.4; Figure 3). Specifically, the lack of allocation concealment and blinding in several studies may have introduced performance and detection bias, contributing to the heterogeneity in the pooled estimates.

#### 4.1.1 Mechanisms by which HIIT improves vascular function indicators in sedentary populations

Sedentary behavior is a major risk factor for cardiovascular disease, negatively affecting vascular health, blood pressure, arterial elasticity, and autonomic regulation (Ekelund et al., 2016). HIIT, due to its efficiency, short duration, and broad applicability, offers unique advantages for improving circulatory function in sedentary populations. Findings from the included studies, consistent with prior research, confirm the beneficial effects of HIIT on endothelial function, blood pressure, PWV, and HR, reinforcing the reliability of these results. The mechanisms by which HIIT improves circulatory indicators in sedentary populations can be summarized in four aspects.

##### 4.1.1.1 Regulation of systolic and diastolic blood pressure

HIIT regulates SBP and DBP through multiple mechanisms. High-intensity exercise enhances parasympathetic activity while suppressing sympathetic tone, contributing to blood pressure reduction (Shi et al., 2024). HIIT also improves insulin sensitivity and promotes glucose and lipid metabolism, indirectly reducing the risk of atherosclerosis and supporting long-term blood pressure control (Shahiddoust and Monazzami, 2025). In addition, HIIT facilitates reductions in body weight and fat mass, further contributing to blood pressure reduction (Delgado-Floody et al., 2020). Evidence suggests that as little as 6 weeks of HIIT can effectively reduce SBP and DBP in sedentary individuals, with greater reductions in SBP compared to equivalent volumes of moderate-intensity continuous training (MICT) (Gonçalves et al., 2024).

##### 4.1.1.2 Improvement of pulse wave velocity (PWV) and arterial elasticity

Pulse wave velocity (PWV) is a sensitive marker of arterial stiffness and directly reflects vascular elasticity. Sedentary behavior is associated with elevated PWV and increased cardiovascular risk (Alansare et al., 2020). High-intensity interval training (HIIT) has been shown to reduce PWV and improve arterial elasticity through multiple synergistic biological mechanisms. First, HIIT promotes vascular structural remodeling by enhancing elastin and collagen metabolism in the arterial wall (Maurer and Clayton, 2022; Shi et al., 2022). Specifically, it promotes elastin synthesis and repair while regulating matrix metalloproteinase activity, which reduces abnormal collagen accumulation and cross-linking (Wang et al., 2021; Fu et al., 2024). Second, HIIT-induced hemodynamic shear stress activates endothelial nitric oxide synthase (eNOS), increases nitric oxide bioavailability, and enhances endothelium-dependent vasodilation, thereby counteracting vascular stiffening (El Karsh, 2021; Srejovic et al., 2023). Furthermore, HIIT attenuates systemic inflammation by reducing inflammatory cytokine levels, which mitigates inflammation-induced endothelial injury and collagen cross-linking, thereby slowing the progression of arterial stiffness (Munk et al., 2011). In summary, through coordinated effects on vascular structure, endothelial function, and inflammatory pathways, HIIT effectively reduces PWV and enhances arterial elasticity. Moreover, HIIT alleviates endothelial injury and inflammation, thereby slowing the progression of arterial stiffening (Boff et al., 2019; Königstein et al., 2022). Multiple experimental studies have shown that 8–12 weeks of HIIT significantly reduces PWV and improves arterial elasticity (Way et al., 2020; Melo et al., 2021).

##### 4.1.1.3 Mechanisms underlying the improvement of endothelial function

HIIT enhances nitric oxide (NO) synthesis in the vascular endothelium, promotes vasodilation, and markedly improves endothelial function. During exercise, increased shear stress stimulates endothelial nitric oxide synthase (eNOS) expression and activity, thereby improving endothelium-dependent vasodilation (Man et al., 2020; Li et al., 2023). Furthermore, HIIT upregulates antioxidant enzyme activity, reduces oxidative stress, and mitigates endothelial damage (Freitas et al., 2018). Some studies have found that HIIT suppresses inflammatory responses and reduces the release of pro-inflammatory mediators, further protecting the endothelium (Allen et al., 2017; Zhang et al., 2024). These synergistic mechanisms render HIIT particularly effective in improving endothelial function in sedentary populations. Additionally, comparative studies between HIIT and MICT in obese male college students showed that both interventions significantly improved endothelial function after 8 weeks of training. However, the mean reactive hyperemia index (RHI) was significantly higher in the HIIT group (Shi et al., 2024).

#### 4.1.2 Mechanisms by which HIIT regulates heart rate in sedentary populations

HIIT reduces resting HR and increases HR variability, reflecting improved cardiac autonomic regulation (Coswig et al., 2020; Navarro-Lomas et al., 2022). It increases vagal tone and decreases sympathetic activity, reducing cardiac workload at rest (Coretti et al., 2025). Moreover, HIIT increases stroke volume, strengthens myocardial function, and improves cardiorespiratory fitness (Crozier et al., 2018; Kumar et al., 2024). Evidence indicates that HIIT lowers both resting and maximal HR, accelerates recovery, and enhances cardiovascular adaptability in sedentary individuals (Schmitz et al., 2018; Songsorn et al., 2022). Recent studies also show that polygenic risk scores, baseline exercise HR, sex, and age can predict variability in HR response to HIIT, providing evidence for personalized exercise interventions (Yang et al., 2023).

#### 4.1.3 Further exploration of the heterogeneity in FMD outcomes

The subgroup analysis identified the participants’ mean age as a primary factor contributing to the heterogeneity of flow-mediated dilation (FMD) outcomes. Notably, a high degree of heterogeneity (I^2^ = 79%) was observed among younger participants (≤30 years). To further clarify the potential heterogeneity factors, we conducted additional exploratory subgroup analyses based on factors such as gender, baseline health status, and comorbidities.

However, none of these additional subgroup analyses showed a significant effect on heterogeneity (data not shown). This “negative finding” may be explained by several factors: ① Data limitations: The original studies reported these variables inconsistently or incompletely, resulting in insufficient data for detailed subgroup analyses and, consequently, limited statistical power. ② Dominant age effect: In sedentary populations, age-related vascular changes may exert such a strong influence that they mask the independent effects of other variables within the available data. As individuals age, the decrease in circulating endothelial progenitor cells and the increase in arterial stiffness cause systematic changes in the pattern and magnitude of vascular responses to exercise interventions (Gollie and Sen, 2022; Lan et al., 2023). Our findings indicate that, in sedentary individuals, age is likely a more decisive factor than sex or baseline health status in determining the efficacy of high-intensity interval training (HIIT) for improving vascular endothelial function. ③ Population homogeneity: Although all studies in this meta-analysis involved sedentary populations, most participants were relatively healthy, and few studies included individuals with severe comorbidities such as clinically diagnosed hypertension or diabetes. This relatively homogeneous baseline health profile may have constrained our ability to detect significant differences among subgroups defined by comorbid conditions. Beyond age-related factors, the considerable heterogeneity observed in the younger subgroup (≤30 years; I^2^ = 79%) may be largely attributed to marked variations in HIIT protocols among the four studies in this category (see Tables 1 and 2). For example, these studies implemented either sprint interval training (SIT) protocols featuring all-out sprints (Jalaludeen et al., 2020) or traditional aerobic interval training (AIT) protocols conducted at higher intensities relative to VO_2_max or HRmax (Huang et al., 2025). These fundamentally different training stimuli—SIT emphasizing neuromuscular and anaerobic adaptations, and AIT focusing on cardiopulmonary and aerobic adaptations—likely induce distinct physiological responses and temporal patterns of endothelial adaptation. Compared with older adults, whose vascular responses tend to be constrained by age-related physiological decline, younger individuals with greater physiological plasticity may show greater variability in FMD responses to these different training stimuli. The interaction between participant age and training protocol type represents an important direction for future research to clarify the sources of heterogeneity and optimize exercise prescription.

#### 4.1.4 Influence of HIIT protocol variations on outcomes

The studies included in this meta-analysis showed substantial differences in their HIIT protocols (Table 2), which may partly explain the high heterogeneity observed for certain outcomes, such as flow-mediated dilation (FMD).

First, with respect to exercise intensity, most studies prescribed 75%–95% of maximal heart rate (HRmax), whereas others used maximal oxygen uptake (VO_2_max) or an “all-out sprint.” These inconsistencies in defining intensity directly affect the absolute workload imposed during training.

Second, the structure of the intervals varied widely, with high-intensity bouts lasting from 10 s to 4 min. These durations correspond to two primary modalities—sprint interval training (SIT) and aerobic interval training (AIT)—which may elicit distinct physiological adaptations (Bidhuri et al., 2025).

Notably, the subgroup analysis of FMD revealed substantial heterogeneity (I^2^ = 79%) among studies with a mean participant age of 30 years or younger. Examination of these studies showed that they employed both the traditional 4 × 4 min AIT protocol and short-sprint SIT protocols. Evidence indicates that different HIIT modalities may vary in both their underlying mechanisms and their efficacy in enhancing endothelial function (Fuertes et al., 2023). Therefore, an interaction likely exists between participant age and the HIIT protocol type, as younger individuals may respond differently to distinct training modalities—potentially contributing to the observed heterogeneity.

Additionally, differences in intervention duration (ranging from 4 to 12 weeks) and per-session workload may influence the magnitude of cumulative adaptations. Therefore, the substantial heterogeneity observed in parameters such as flow-mediated dilation (FMD) may stem not only from participant characteristics but also from variations in the high-intensity interval training (HIIT) protocols used across the included studies. Despite these variations, the meta-analysis consistently demonstrated that HIIT has positive effects on cardiovascular parameters, suggesting its general applicability as an effective training approach. Future studies and clinical practice should prioritize the standardization and detailed reporting of HIIT protocol parameters to identify which approaches are most effective for specific populations and target outcomes.

#### 4.1.5 Interpretation of PWV results

The robustness of our findings regarding the effect of HIIT on pulse wave velocity (PWV) is limited. This was demonstrated by the loss of statistical significance when applying a more conservative random-effects model to account for moderate heterogeneity (I^2^ = 47%). Critically, sensitivity analysis confirmed this instability, revealing that the pooled result was heavily dependent on the inclusion of a single study (Huang et al., 2025). This over-reliance on one study is fundamentally attributable to the small number of studies included in the analysis (only four). With such a limited evidence base, the influence of any individual study with a distinct effect size—potentially due to unique participant characteristics, specific HIIT protocol parameters, or PWV measurement methodologies—becomes disproportionately large, thereby destabilizing the overall pooled estimate. The clear implication is that while a statistically significant improvement in PWV was observed under a fixed-effects model, this conclusion is fragile. Therefore, the evidence for HIIT improving arterial stiffness in sedentary populations is less robust compared to the findings for blood pressure and endothelial function, which are supported by a larger and more consistent body of evidence. This outcome should be interpreted as preliminary and highlights the need for future high-quality studies with larger sample sizes to provide a more definitive and stable estimate.

### 4.2 Strengths and limitations

#### 4.2.1 Strengths

This systematic review demonstrates several significant strengths. First, to the best of our knowledge, this is the first meta-analysis focusing exclusively on sedentary individuals and comprehensively evaluating the effects of high-intensity interval training (HIIT) on multiple cardiovascular outcomes—including blood pressure, arterial stiffness, endothelial function, and heart rate—thus addressing a critical gap in the existing evidence base. Second, the study followed rigorous methodological standards, including prospective registration in the PROSPERO database, strict adherence to the PRISMA reporting guidelines, a comprehensive bilingual search strategy (English and Chinese databases), and independent screening and quality assessment by two reviewers, thereby ensuring methodological transparency and reliability of the results. Third, an in-depth subgroup analysis was performed to investigate the substantial heterogeneity observed in flow-mediated dilation (FMD), identifying age as a potential moderating factor. These findings offer valuable insights into population-specific differences in HIIT responses.

#### 4.2.2 Limitations

Notwithstanding these strengths, several limitations warrant consideration. ① Grey literature and studies published in languages other than Chinese and English were excluded, which may have led to the omission of relevant studies. ② Although all participants were categorized as sedentary, some had comorbid type 2 diabetes or cardiovascular risk factors, which could have introduced bias. ③ Variations in HIIT protocols, exercise intensity, and intervention duration among the included studies may have contributed to inter-study heterogeneity. ④ The absence of original data for some outcome measures may have reduced the accuracy of the estimated effect sizes. ⑤ Although the funnel plot analysis indicated no obvious publication bias, its potential presence cannot be completely ruled out. ⑥ The conclusions regarding PWV in this study were based on a limited number of trials, and the point estimates exhibited fluctuations in the sensitivity analysis, suggesting that the stability of this effect warrants further confirmation in future research. ⑦ This meta-analysis employed several standard statistical methods during data synthesis, including imputing missing change standard deviations by assuming R = 0.5. While these methods are commonly applied, their potential influence on the results should be recognized.

### 4.3 Long-term efficacy, safety, and future directions

#### 4.3.1 Gaps in long-term efficacy and safety considerations

Most clinical trials included in this study had short intervention periods (typically 8–12 weeks) and lacked long-term follow-up data. Although existing evidence clearly shows that HIIT significantly improves circulatory parameters during the intervention period, the long-term maintenance of these benefits remains unclear. Because sedentary behavior constitutes a long-term lifestyle pattern, assessing both adherence to and maintenance of exercise-induced effects is essential. Currently, few studies have examined how long the beneficial effects of HIIT on blood pressure, vascular elasticity, and endothelial function persist after the intervention. This lack of evidence on long-term effects limits the ability to comprehensively evaluate HIIT’s potential as a sustainable public health intervention. While HIIT is widely recognized for its effectiveness, its potential risks should not be underestimated—especially among sedentary individuals with increased cardiovascular risk. Although the randomized controlled trials included in this study did not report any serious adverse events, this finding may be explained by the exclusion of participants with severe cardiovascular disease, the use of supervised exercise settings, and relatively small sample sizes. HIIT can induce rapid, short-term elevations in heart rate and blood pressure, which may theoretically precipitate cardiovascular events such as arrhythmias or myocardial ischemia. For individuals with undiagnosed or subclinical cardiovascular conditions—which are relatively prevalent among sedentary populations—medical screening and risk assessment before initiating high-intensity exercise are essential. Therefore, when promoting HIIT among sedentary populations, it is crucial to emphasize progressive adaptation and individualized program design. For middle-aged and older adults, as well as individuals with known cardiovascular risk factors, training should begin under professional supervision and medical oversight.

#### 4.3.2 Future research directions

Building on the findings of this study and the preceding discussion, future research should address the following areas: ① Long-term follow-up studies: Randomized controlled trials with longer intervention durations (e.g., ≥6 months) and post-intervention follow-ups are urgently required. These studies are crucial to assess the persistence and decline of HIIT benefits and to identify the optimal training frequency and intensity needed to maintain these health gains. ② Safety and special population studies: Future research should systematically monitor and report adverse events while intentionally recruiting sedentary participants with varying cardiovascular risk profiles. This approach can help define the safety margins and efficacy limits of HIIT across different risk levels and guide the development of safe and evidence-based exercise prescriptions. ③ Personalized and optimized protocols: Future studies should identify HIIT protocols that best balance efficacy, safety, and adherence. Given this study’s indication that age may contribute to heterogeneity, future research should further investigate how age, sex, and baseline health status influence individual responses to HIIT, thereby supporting the development of precision exercise prescriptions. ④ Combined intervention strategies: Future research should examine whether combining HIIT with other lifestyle interventions can produce synergistic effects, thereby providing sedentary populations with more comprehensive and sustainable strategies for health promotion.

## 5 Conclusion

In summary, this meta-analysis demonstrates that high-intensity interval training (HIIT) effectively enhances key cardiovascular outcomes in sedentary individuals, producing significant improvements in blood pressure, vascular elasticity, and endothelial function. Therefore, HIIT should be recognized as an effective and practical exercise strategy for improving vascular health in sedentary populations. Given the limitations of existing evidence, additional well-designed, long-term clinical trials are required to confirm the sustained efficacy and safety of HIIT and to facilitate its individualized and precision-based applications. Considering the substantial heterogeneity observed in flow-mediated dilation (FMD) outcomes among younger participants and the sensitivity of pulse wave velocity (PWV) results, future studies should focus on standardizing HIIT variables such as training intensity, interval structure, and intervention duration to improve comparability across studies. Moreover, long-term follow-up should be implemented to confirm the durability of these circulatory improvements.

## Data Availability

The original contributions presented in the study are included in the article/supplementary material, further inquiries can be directed to the corresponding author.
